# Age-Related Increases in Verbal Knowledge Are Not Associated With Word Finding Problems in the Cam-CAN Cohort: What You Know Won’t Hurt You

**DOI:** 10.1093/geronb/gbw074

**Published:** 2016-06-30

**Authors:** Meredith A. Shafto, Lori E. James, Lise Abrams, Lorraine K. Tyler, Cam-CAN

**Affiliations:** ^1^Centre for Speech, Language and the Brain, Department of Psychology, University of Cambridge, UK.; ^2^Department of Psychology, University of Colorado Colorado Springs.; ^3^Department of Psychology, University of Florida, Gainesville.

**Keywords:** Word retrieval, Naming, Tip-of-the-tongue (TOT), Knowledge, Interference

## Abstract

**Objective:**

We tested the claim that age-related increases in knowledge interfere with word retrieval, leading to word finding failures. We did this by relating a measure of crystallized intelligence to tip-of-the-tongue (TOT) states and picture naming accuracy.

**Method:**

Participants were from a large (*N* = 708), cross-sectional (aged 18–88 years), population-based sample from the Cambridge Centre for Ageing and Neuroscience cohort (Cam-CAN; www.cam-can.com). They completed (a) the Spot-the-Word Test (STW), a measure of crystallized intelligence in which participants circled the real word in word/nonword pairs, (b) a TOT-inducing task, and (c) a picture naming task.

**Results:**

Age and STW independently predicted TOTs, with higher TOTs for older adults and for participants with lower STW scores. Tests of a moderator model examining interactions between STW and age indicated that STW was a significant negative predictor of TOTs in younger adults, but with increasing age, the effect size gradually approached zero. Results using picture naming accuracy replicated these findings.

**Discussion:**

These results do not support the hypothesis that lifelong knowledge acquisition leads to interference that causes an age-related increase in TOTs. Instead, crystallized intelligence supports successful word retrieval, although this relationship weakens across adulthood.

One of the most commonly reported complaints of old age is the increase in temporary word finding failures known as tip-of-the-tongue (TOT) states. Although TOTs feel like serious memory failures, research supports the phonological retrieval deficit (PRD) hypothesis, which suggests that TOTs reflect temporary failures accessing phonological (word form) representations during word retrieval. Under the PRD hypothesis, TOTs become more frequent as we get older because the connections between semantic (word meaning) and phonological representations weaken with age (Burke, MacKay, & James, 2000; Burke, MacKay, Worthley, & Wade, 1991). An alternative account focuses on interference or blocking (Jones, 1989), where TOTs reflect failures to activate a target word due to interference from related concepts. Some researchers have suggested that age-related increases in word knowledge increase interference among known words, leading to older adults’ more frequent word finding failures (Dahlgren, 1998; Ramscar, Hendrix, Shaoul, Milin, & Baayen, 2014). The current study tested these two accounts of age-related word finding failures by examining the relationship between TOTs and crystallized intelligence in a large, cross-sectional, population-based sample of participants aged 18–88 years.

Whereas some cognitive functions decline with age, general knowledge, vocabulary, and other measures of crystallized knowledge are stable or improve across the life span (Salthouse, 2009, 2010, 2014). Lifelong knowledge acquisition leads to a more enriched and interconnected network of conceptual semantic information that could lead to age-related increases in semantic interference (Laver & Burke, 1993; Taylor & Burke, 2002). This account predicts that age-related increases in semantic interference exacerbate retrieval difficulty, resulting in more frequent TOTs. However, evidence from previous research is limited and mixed. Dahlgren (1998) provides the only finding using a TOT experiment that increased vocabulary knowledge is associated with increased TOT rates. Participants generated TOTs in response to general knowledge questions, and participants with a higher proportion of TOTs had higher Wechsler Adult Intelligence Scale–Revised vocabulary scores (Wechsler, 1981), even when age was factored out. More recently, Ramscar et al. (2014) claimed that evidence from a series of simulations demonstrates that increased knowledge can lead to increased interference. However, Ramscar et al.’s simulations largely do not reflect TOT or other production tasks, including simulations of lexical decision, letter classification, paired associate learning, and name recognition. The single simulation of a production task (phonemic fluency) in that paper showed that age-related experience improved performance. Other recent studies have found no relationship between vocabulary and age-related increases in TOTs (Facal, Juncos-Rabadán, Rodríguez, & Pereiro, 2012) or found that age-related impairment persists when vocabulary is matched between younger and older adults (Juncos-Rabadán, Facal, Rodríguez, & Pereiro, 2010). Finally, Kavé and Yafé (2014) found that within both older and younger groups, measures of vocabulary were associated with improved naming and verbal fluency, suggesting that vocabulary knowledge supports rather than interferes with production.

In contrast to the interference account, the PRD hypothesis suggests that age-related increases in TOTs are due to age-related phonological access deficits (Burke et al., 1991, 2000). The PRD hypothesis can also account for other age-related changes in lexical production, including increases in dysfluencies in natural speech, increased picture naming errors (Burke & Shafto, 2008), and declines in phonological access during picture naming (Taylor & Burke, 2002). The PRD hypothesis predicts that increased verbal knowledge should reduce the prevalence of TOTs by increasing phonological support for the target word. Increasing the number of known words creates denser “neighborhoods” of words that are phonologically similar, supporting faster and more accurate phonological access during production (Vitevitch, 2002; Vitevitch & Sommers, 2003). Words from dense phonological neighborhoods elicit fewer TOTs in both younger and older adults (Harley & Macandrew, 2001; Vitevitch & Sommers, 2003). Similarly, older (but not younger) adults have fewer TOTs for words with high-frequency first syllables (Farrell & Abrams, 2011). If older adults’ greater crystallized knowledge gives them more words with denser phonological neighborhoods and high first-syllable frequency, this should attenuate increases in word finding failures.

The current study examined the relationship between crystallized intelligence and word retrieval using the Cambridge Centre for Ageing and Neuroscience (Cam-CAN; www.cam-can.com) cohort. Our primary measure of word retrieval failures was TOTs for public figures, as our task used proper names as target stimuli. Some models suggest that proper nouns may be stored and retrieved differently than common nouns (Semenza, 2006, 2009) and therefore may not provide the best test of the interference hypothesis. To test the generalizability of our findings from TOTs for proper names to word finding problems for common nouns, we repeated key analyses using data from a picture naming task where participants named familiar objects out loud. We expected that age and crystallized intelligence will have similar effects on both TOTs and picture naming accuracy.

Finally, previous examinations relating TOTs to vocabulary or general knowledge have relied on volunteer cohorts (Dahlgren, 1998) or simulations (Ramscar et al., 2014). The Cam-CAN cohort is population-based and thus avoids issues that may accompany the use of younger participants who are university students or older volunteers who may be unrepresentative of the general population. Moreover, the Cam-CAN cohort is adequately large (*N* > 700) to allow us to observe changes in the relationship between crystallized intelligence and TOTs across the adult life span.

## Method

### Participants

Participants were 708 healthy adults aged 18–88 years who took part in cognitive testing as part of the population-based Cam-CAN cohort (see Shafto et al., 2014, for full protocol details). For some analyses, participants were divided into groups of younger, middle-aged, and older adults (see Table 1, for age ranges). Participants were recruited equally across seven deciles from age 18 to 87, and equal numbers of men and women were recruited within each decile (50.7% female). For details of recruitment including the sampling frame and exclusion criteria, see Shafto and coworkers (2014). Across the sample, 60% of participants had university education (75% of younger adults, 64% of middle-aged adults, and 47% of older adults). We defined educational attainment as a binary measure of university degree attainment rather than a continuous measure in order to capture meaningful categorical transitions such as university degrees that mark levels of qualification.

**Table 1.** Descriptive Data and Correlations with Age for Key Variables

		*N*	*M*	Range	*SD*	Age correlation
Age	All	708	54.64	18–88	18.63	
	Younger	178	30.25	18–39	5.82	
	Middle	280	51.71	40–64	7.33	
	Older	250	75.29	65–88	6.17	
TOT ratio	All	644	.46	0–1.00	.24	.31**
	Younger	155	.39	0–.84	.20	−.09
	Middle	266	.42	0–1.00	.21	.06
	Older	223	.56	0–1.00	.26	.24**
Picture naming accuracy (proportion correct)	All	648	.78	.50–.94	.09	−.55**
	Younger	160	.83	.59–.94	.06	.32**
	Middle	264	.81	.54–.93	.07	−.29**
	Older	224	.71	.50–.89	.08	−.33**
STW total (out of 60)	All	705	53.58	24–60	5.39	.22**
	Younger	177	51.56	24–60	5.60	.29**
	Middle	280	54.23	30–60	4.50	.08
	Older	248	54.28	29–60	5.79	.17**
Cattell total (out of 46)	All	660	31.80	11–44	6.79	−.66**
	Younger	168	37.14	22–44	4.27	.02
	Middle	263	33.24	18–43	4.95	−.28**
	Older	229	26.22	11–40	6.09	−.35**

**p < .01.

### Materials and Procedure

We used measures included in an extensive program of epidemiological and neurocognitive testing described in Shafto and coworkers (2014). For the TOT task, participants viewed 50 faces of public figures (e.g., actors, politicians, and athletes) and had 5s per face to respond. Participants named each person (Know response), said they did not know the name (Don’t Know response), or said they were having a TOT. Faces were included that had been previously pretested with (*N* = 9) young and (*N* = 11) older adults to avoid floor effects (i.e., high Don’t Know rates). We report TOTs as a ratio of attempted responses (TOTs / [TOTs + Know responses]), in keeping with previous research (e.g., Gollan & Brown, 2006). For the crystallized intelligence task, we used the Spot-the-Word Test (STW; Baddeley, Emslie, & Nimmo-Smith, 1993). STW was designed to measure premorbid intelligence and assesses verbal intelligence using lexical decision. Baddeley and coworkers (1993) established the validity of STW in relating to crystallized abilities, independent of fluid abilities. The task was paper-and-pencil, where participants had 5min to view word/nonword pairs and circle the real word. Correct responses were scored 1 point, for a maximum score of 60. Finally, we used a picture naming task to provide a measure of common noun retrieval. Participants viewed 200 pictures of common objects and had 1,750ms per picture to provide the name. Object images were selected from the Hemera picture database (Hemera Technologies, Canada). Name agreement and familiarity ratings were determined in a pretest with *N* = 16 younger adults, and pictures had a mean name agreement of 91% (*SD* = 10%) and a mean familiarity rating (7-point scale) of 5 (*SD* = .92). Naming accuracy was measured as proportion correct.

We included two covariates in all regression analyses: university education and fluid intelligence. Because educational attainment likely relates to crystallized intelligence, university education was included as a binary predictor. We used fluid intelligence in order to control for general intellectual ability. We used the standard form of the Cattell Culture Fair, Scale 2 Form A (referred to here as Cattell; Cattell & Cattell, 1973), a paper-and-pencil task comprised of four subtests with different types of nonverbal puzzles. Correct responses were given a score of 1 for a maximum total score of 46. Finally, continuous interactions between age and STW were examined using moderator models to test whether the predictive effect of crystallized knowledge changed across the age range. Significant interactions were followed up using the Johnson–Neyman technique (Johnson & Fay, 1950) that provides regions of significance (i.e., age ranges where STW was a significant predictor).

## Results

The effect of age on TOTs and STW can be seen in the binary correlations with age and in mean performance within age groups (see Table 1). In keeping with previous findings, age was associated with increasing TOTs (Burke et al., 1991). Age was also associated with increasing STW performance. A previous examination found no effect of age on STW (Yuspeh, 2000) but was in a limited age range of only older adults; to our knowledge, STW has not previously been examined across such a wide age range.

The relationship of crystallized intelligence (STW) to TOTs was examined in linear regressions, which also included age, Cattell, and university education. Table 2 shows the results for all participants and for younger, middle-aged, and older adults separately. Across all participants, age and crystallized intelligence independently predicted TOTs, with higher TOTs for older adults and participants with lower crystallized intelligence. Note that including STW as a predictor strengthened the effect of age (*t* = 4.80, *p* <.001; also see Table 2) compared with the effect of age in a similar regression that did not include STW (*t* = 3.83, *p* < .001).

**Table 2:** Linear Regressions of Variables Predicting TOT Ratio and Picture Naming Accuracy

		TOT Ratio	Picture Naming Accuracy
Age	All	.003** (.002, .005)	−.002** (−.002, −.002)
	Younger	−.001 (−.007, .005)	.002* (.000, .004)
	Middle	.001 (−.002, .005)	−.002** (−.003, −.001)
	Older	.012** (.006, .018)	−.004** (−.006, −.002)
STW total	All	−.007** (−.011, −.003)	.003** (.002. .004)
	Younger	−.011* (−.020, −.002)	.005** (.003, .007)
	Middle	−.007* (−.013, −.001)	.002* (.000, .004)
	Older	−.008* (−.015, −.001)	.002* (.000, .004)
Cattell total	All	−.004^†^ (−.007, .000)	.002** (.001, .004)
	Younger	−.006 (−.014, .003)	−.001 (−.004, .001)
	Middle	−.003 (−.008, .003)	.003** (.001, .004)
	Older	.000 (−.007, .007)	.002* (.000, .004)
University education	All	−.030 (−.072, .012)	.009 (−.003, .022)
	Younger	−.045 (−.129, .039)	.018 (−.006, .041)
	Middle	−.046 (−.106, .014)	−.001 (−.019, .018)
	Older	−.002 (−.083, .079)	.008 (−.015, .031)

*Notes:* Beta coefficients with 95% confidence intervals are given in parentheses. STW = Spot-the-Word Test; TOT = tip-of-the-tongue.

^†^ *p* < .09. *p < .05. **p < .01.

**Figure 1.**
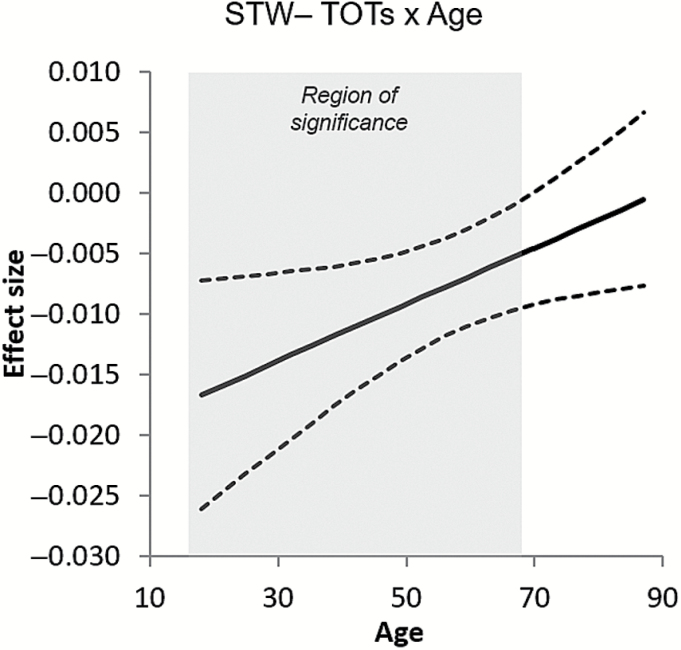
Results of a moderation model examining the continuous interaction of age with the effect of STW on TOT ratio. Solid lines show the effect size, dashed lines indicate 95% confidence intervals, and the region of significance is indicated by the gray area. STW = Spot-the-Word Test; TOT = tip-of-the-tongue.

When participants were divided into three age groups, the effect of STW on TOTs was significant for young, middle-aged, and older adults separately (see Table 2). However, the effect was strongest in younger adults and weaker in middle-aged and older groups. We examined this apparent age difference in a moderator model testing for continuous interactions between STW and age (Hayes, 2013). Table 3 shows the main effects of STW and a significant interaction between STW and age. Figure 1 illustrates how the effect size relating STW and TOTs gradually changes across the age range. The Johnson–Neyman technique (Johnson & Fay, 1950) was used to provide regions of significance for the relationship between STW and TOTs. The region of significance shown in Figure 1 demonstrates that STW is a significant negative predictor in younger adults, but with increasing age, the effect size gradually approaches zero until it reaches nonsignificance at approximately age 69.

**Table 3:** Results of Moderator Models Evaluating the Interaction of STW and Age in Predicting TOT Ratio and Picture Naming Accuracy

	TOT Ratio	Picture Naming Accuracy
Age	−.010 (−.021, .002)	.004* (.001, .007)
STW	−.022** (−.034, −.009)	.009** (.006, .012)
Age × STW	.0002* (.0000, .0005)	−.0001** (−.0002, −.0001)
Cattell	−.004^†^ (−.007, .000)	.002** (.001, .003)
University education	−.010 (−.037, .009)	.008 (.001, .015)

*Notes:* Beta coefficients with 95% confidence intervals are given in parentheses. STW = Spot-the-Word Test; TOT = tip-of-the-tongue.

^†^ *p* < .09. *p < .05. **p < .01.

These results do not support the hypothesis that increased word knowledge causes interference during lexical retrieval. To test whether the findings from proper names generalize to common nouns, we repeated key analyses using picture naming accuracy as the outcome measure. Key results from TOT analyses were replicated: First, naming accuracy declined with age (see Table 1). Second, STW significantly predicted naming accuracy in a regression (see Table 2), and the effect of age was stronger with STW included (*t* = −9.41, *p* < .001) than when STW was removed (*t* = −7.67, *p* < .001). Third, a moderator model demonstrated that the relationship between STW and naming accuracy was strongest in younger adults and declined with age (see Table 3), with a Johnson–Neyman test providing a region of significance ending at approximately age 70.

## Discussion

The current study examined the link between crystallized intelligence and TOTs in a large, adult life span, population-based sample of healthy adults. Results demonstrate that age and crystallized intelligence are independent predictors of TOTs for proper names, with fewer TOTs associated with higher STW and youth, despite an overall positive relationship between age and STW. These relationships remain when measures of general intellectual ability (fluid intelligence) and educational attainment are factored out, and similar relationships were found for picture naming accuracy. These results argue against the hypothesis that age-related increases in word finding problems are due to age-related increases in acquired verbal knowledge (e.g., Ramscar et al., 2014).

Indeed, significant relationships between STW and TOTs were in the opposite direction from predictions of an interference hypothesis. Moreover, this relationship weakened rather than strengthened with age, both for TOTs and picture naming accuracy. The direction of the relationship of STW and picture naming accuracy is in keeping with studies relating verbal knowledge to naming such as Kavé and Yafé (2014) who reported that vocabulary was positively associated with better naming performance. However, our findings are not in keeping with those of Dahlgren (1998) who found that age-related increases in vocabulary knowledge were associated with higher TOT rates. It is difficult to know whether the difference in findings may be due to methodological choices that differed from ours, including the type of TOT materials (general knowledge questions) or vocabulary assessment (producing definitions of words); as we note in the Introduction, another possible difference is the use of a volunteer sample in Dahlgren’s study, whereas the Cam-CAN recruitment was population-based.

Our findings are also not in line with the hypothesis of Ramscar and coworkers (2014) that age-related knowledge acquisition routinely leads to increased interference in language processing. As noted in the Introduction, it is likely that our findings differ from those of Ramscar and coworkers because the simulations in that study do not focus on processes that underpin age-related changes in lexical retrieval during production. In particular, many of their simulations were of language perception/comprehension tasks (e.g., lexical decision and name recognition) that are typically well preserved in old age; a number of studies demonstrate that the effect of age can reverse when examining language production compared with perception/comprehension tasks (James & MacKay, 2007; MacKay & Abrams, 1998; MacKay, Abrams, & Pedroza, 1999; Shafto, 2010). As to why Ramscar and coworkers’ simulations suggest age-related interference during comprehension, there are specific situations in which age-related increases in lexical knowledge could lead to increased semantic interference. Laver and Burke (1993) addressed this possibility in a meta-analysis. They suggest that older adults’ greater knowledge leads to a more enriched and interconnected semantic network so that related concepts may interfere more during semantic priming tasks. This account is not identical to Ramscar and coworkers’ suggestion that the number of learned words causes interference but is in keeping with the results of, for example, the simulated lexical decision data suggesting slower responses for the “older” model with increased knowledge (Ramscar et al., 2014, simulation study 2).

The current results are more consistent with the PRD hypothesis that predicts that additional word knowledge creates denser phonological neighborhoods that support phonological retrieval during production. Although the relationship between STW and TOT weakened with age, there was some indication that STW differentially supports word retrieval in older adults, as covarying out STW strengthened the age-related declines of both TOTs and picture naming accuracy. This suggests researchers may be *underestimating* the age-related decline in phonological access. However, our finding that STW becomes a weaker predictor of word retrieval in old age is not clearly explained by the PRD hypothesis. There is evidence that phonological neighborhoods develop as childhood vocabulary increases (Charles-Luce & Luce, 1990) and that words acquired earlier tend to be more common and part of denser neighborhoods, whereas words acquired later add less to neighborhoods because they are lower frequency and more phonologically isolated (Coady & Aslin, 2003). Future research should explore whether the link between verbal knowledge and phonological neighborhood density extends into adulthood and examine whether such a link decreases with age in keeping with the weakening relationship between STW and TOTs.

In conclusion, the current results demonstrate that age-related increases in word finding failures are not associated with lifelong increases in verbal knowledge. Indeed, our findings raise the possibility that age-related changes in word finding failures have previously been underestimated by not taking into account the potentially compensatory role of increased lexical knowledge in older adults’ word retrieval. Finally, our results highlight the importance of employing well-specified models of language and aging in order to avoid overly general assumptions that aging affects language production and comprehension in monolithic ways.

## Funding

The Cambridge Centre for Ageing and Neuroscience (Cam-CAN) research was supported by the Biotechnology and Biological Sciences Research Council (grant number BB/H008217/1).
